# Does familiarity with an idea bias its evaluation?

**DOI:** 10.1371/journal.pone.0286968

**Published:** 2023-07-05

**Authors:** Anne Greul, Tim G. Schweisfurth, Christina Raasch

**Affiliations:** 1 Independent researcher, Munich, Germany; 2 Institute for Organizational Design and Collaboration Engineering, Hamburg University of Technology, Hamburg, Germany; 3 Kühne Logistics University, Hamburg, Germany; 4 Kiel Institute for the World Economy, Kiel, Germany; The Hong Kong Polytechnic University, HONG KONG

## Abstract

Although many organizations strive for radical or disruptive new ideas, many fall short of their goals. We propose that a primary reason for this failure is rooted in the individuals responsible for innovation: while they *seek* novel ideas, they *prefer* familiar ones. While prior research shows that individuals are biased against ideas with high *objective novelty*, it has overlooked the role of *subjective novelty*, i.e., the extent to which an idea is novel or unfamiliar to an individual idea evaluator. In this paper, we investigate how such subjective familiarity with an idea shapes idea evaluation in innovation. Drawing on research from psychology and marketing on the *mere exposure effect*, we argue that familiarity with an idea positively affects the evaluation’s outcome. We present two field studies and one laboratory study that support our hypothesis. This study contributes to the understanding of cognitive biases that affect innovation processes.

## Introduction

*The genesis of one of the world’s most essential drugs coincided with the discovery of one of the evilest ones. In 1897, the young chemist Felix Hoffman synthesized two drugs in Bayer’s labs in Elberfeld, Germany: acetylsalicylic acid, and eleven days later, diacetylmorphine. He presented both substances to the lab leader, Heinrich Dreser, who was responsible for filling the painkiller pipeline, looking for a substitute for morphine. He rejected the former substance (later known as Aspirin) and picked the latter for further testing, clearing the pathway to market for one of the world’s deadliest substances (branded as Heroin). While it is impossible to retrace exactly why Dreser selected Heroin over Aspirin, it seems likely that familiarity played a key role. Dreser was very familiar with morphine derivatives (such as heroin) and had investigated their effect on the respiratory system. In selecting Heroin over Aspirin, he chose the familiar over the unfamiliar [1]*.

Anything new that is eventually implemented by an organization starts with an idea. Since most ideas turn out to be unpromising, firms need to generate many ideas to find the good ones. With the advent of digital technologies, generating large numbers of ideas has become easy since firms can “crowdsource” idea generation: they openly call for ideas, outsourcing the idea generation task to an unknown crowd within or outside the organization. Crowdsourcing ideation overcomes the challenge of having too few ideas but exacerbates the problem of idea selection: Since only very few ideas can and should be implemented, firms must decide which ones to reject and which ones to select. Evaluating ideas is costly and time-intensive. Faced with this cognitive burden, evaluators are known to resort to heuristics, that is mental shortcuts, leading to biased idea selection. Specifically, the characteristics of the *idea itself may* bias its evaluation. For example, idea novelty [2], idea enactment [3], and idea description [4] have been shown to affect evaluators’ decisions.

In this paper, we propose another important but overlooked mechanism: the decision makers are tasked with seeking novel, but prefer ideas with which they are familiar. This mechanism may be at the root of several organizational pathologies in which ideas are systematically underrated or overrated, resulting in a failure to implement the best ideas. For instance, firms may prefer internal over external ideas [5], since employees have been exposed to internal ideas more often. Also, employees in firms lack the ability to absorb new ideas, because they have lower exposure to ideas from unfamiliar knowledge domains [6]. Finally, organizational myopia may be rooted in the fact that employees prefer well-known ideas to potential breakthrough ideas [7].

The literature on novelty’s role in idea evaluation addresses the novelty of ideas as a collective, consensual, and objective phenomenon [8–11]–as the relationship between an idea and some kind of collective, either a firm [7], a panel [2], or a scientific field [12]. The research has analyzed how this *objective novelty* biases idea evaluations in organizations and disciplines [e.g. 2,13,14]. We will use the term objective novelty here but acknowledge that novelty can never be fully objective since it is based on an audience’s collective perceptions of novelty. We choose the term objective novelty instead of collective novelty to stress the opposites of objective and subjective novelty.

We acknowledge the merits of this perspective but argue that it misses the relationship dimension that connects the idea and the idea evaluator [9,15]. We propose *subjective novelty–*the idea’s degree of novelty to the individual idea evaluator–as a distinct concept. An idea may be familiar (subjectively not novel) to an individual in the organization but novel to the organization, or the other way around. We argue that individual idea evaluators are likely to be guided by their subjective perceptions of novelty.

We focus on familiarity, the opposite of subjective novelty, which allows us to draw on the rich psychological literature on familiarity [16,17] and the so-called *mere exposure effect* [18–20]. The research has shown that familiarity and novelty are not necessarily counterparts on collective level, for instance concerning firm routines [21] or category labels [22]. This is different for the individual level, where less familiar is more novel and vice versa. We argue that familiarity with an idea positively affects idea evaluation independently of true idea quality, i.e. that familiarity increases idea evaluation bias.

To test this hypothesis, we conducted two field studies and one laboratory study. In field study 1, we conducted a factorial survey-based vignette experiment in which 210 employees from a large automotive manufacturer evaluated multiple ideas and also expressed how familiar they were with these ideas. Field study 2 consisted of a within-person experiment, in which we manipulated idea familiarity by selecting one of the ideas for subsequent re-evaluation in the same sample. The field studies involved engineers and marketing specialists who regularly evaluate innovative ideas as part of their jobs, offering high external validity. Results have higher generalizability when they are drawn from a sample of real decision-makers. Study 3, an experimental lab study with 283 students from a technical university, allowed us to overcome the methodological limitations of our field studies and to control for confounding variables by randomizing the treatment, establishing causality. In all these studies, we find that familiar ideas receive excessively positive evaluations, while unfamiliar ideas are penalized.

Our study contributes to the literature on idea evaluation in innovation. We establish subjective novelty (or familiarity as its analog) as a new antecedent of idea evaluation, independent of idea quality and objective novelty. This is significant, since this bias against subjective novelty is likely at the root of a problem that affects innovation generally: individuals are supposed to *seek* the unfamiliar and novel, but *prefer* the known. We show that the idea familiarity level of the individual evaluator (not the organization)–a personal factor–can affect which ideas are developed in organizations and suggest that organizations that aspire to radical innovation take measures to counteract this bias.

## Evaluating ideas in the innovation process

Innovation is a process covering the phases of idea generation, idea selection, and idea implementation. In this paper, we focus on the second phase where ideas are screened, evaluated, and selected. By definition, ideas are novel and only reveal their true value after implementation. However, since organizational resources are limited and not all ideas can be implemented, firms have to decide which ideas to select and which ones to discard, without knowing an idea’s true value: they estimate an idea’s value ex ante and under uncertainty [23].

The uncertain nature of the idea evaluation process renders idea evaluation inaccurate: Since evaluators cannot assess the true value of an idea, they rely on other cues that are available, but that may introduce error and bias into idea evaluation. Scholars have investigated different factors that represent relevant cues in idea evaluation [15]. First, the characteristics of the *idea creator* may systematically affect or bias the idea evaluation process. For example, evaluators prefer ideas that come from known idea creators [24], from individuals hierarchically close to them in the organization [25] and individuals with whom they share a social identity [26]. Second, factors rooted in the idea *evaluator* may introduce error into the idea evaluation process. For example, individuals in decision making roles evaluate differently from other individuals [14,24,27]; individuals higher up in the organizational hierarchy tend to overvalue their own ideas [28]; and individuals’ personality affects how they perceive novel ideas [9]. Third, the idea evaluation *context* affects evaluation outcomes. For example, evaluators adapt their evaluation scores to other evaluators in their social context [29,30]; are more accurate in their idea evaluation in authoritative organizational contexts [31]; and their perceptions of novelty are affected by organizational culture [4,9]. Finally, factors related to the *evaluation target*, i.e., the idea itself, may affect evaluators’ estimations, for example its enactment [3], description [4] or novelty [2]. Our paper speaks to this last strand of research.

## Novelty and familiarity in the evaluation of ideas

### Objective vs. subjective novelty

Objective and subjective evaluations of an idea’s novelty are not the same [10]; they represent independent dimensions [8]. Objective novelty captures whether an idea is new in terms of collectively held standards [9], while subjective novelty expresses whether or not an idea is new to an individual. An idea may be highly novel for an organization (i.e. it has high objective novelty) because it involves a novel recombination of knowledge components. The same idea may be familiar to an individual manager whose team has developed this idea (low subjective novelty). Conversely, an idea can be widely known in an organization but can still be unfamiliar to a particular employee.

According to Amabile’s [10] seminal definition of consensual novelty (which we call objective novelty) [cf. 9], an idea “is creative to the extent that appropriate observers independently agree it is creative” (p. 1001). Similarly, Csikszentmihalyi [11] pointed out that novelty “is not the product of single individuals, but of social systems making judgments about individuals’ products” (p. 102). In this view, subjective perceptions of novelty aggregate to collective, “objective” novelty.

*Objective novelty* has received some attention in the literature on idea evaluation. Boudreau et al. [13] investigated how novelty affects researchers’ evaluations of research proposals, conceptualizing novelty as the relationship between a research proposal and an existing body of research. Criscuolo et al. [2] analyzed how novelty affects panels’ funding of new projects, measuring novelty as the relationship between an idea proposal and a firm’s database of existing projects. Berg [14] and Lu and Bartol [3] assessed novelty’s effects on professionals’ idea evaluations, conceptualizing novelty as what a group of individual judges to be novel. Chai and Menon [12] investigated novelty’s effects on citations, measuring novelty as the distance between keywords in a paper and all keywords in a body of papers in a year. Piezunka and Dahlander [24] show that ideas that are similar in content to ideas that have been suggested to organizations before are more likely to be implemented. In all these studies, novelty was conceptualized as consensual, collective, or objective; it captures the relationship between an idea and some collective standard.

To date, the notion of *subjective novelty* has received very limited attention in the idea evaluation literature. Amabile [10] pointed out the differences between social and subjective creativity. Zhou et al. [9] investigated which personal (e.g. promotion/prevention focus) and contextual (e.g. an innovation culture) factors moderate the relationship between objective and subjective novelty.

We seek to contribute to this research stream by investigating how subjective novelty affects our evaluation of ideas. To be able to build on pertinent literature from psychology, we focused on the conceptual counterpart of subjective novelty–familiarity. We seek to isolate idea familiarity’s biasing effect on idea evaluation, showing it to be independent of objective novelty.

### Familiarity

Psychologists have extensively studied the concept of familiarity and its effects on individual attitudes and behaviors. Most importantly for this study, they found that a stimulus’ familiarity is associated with more positive assessments thereof. This finding is encapsulated in the *mere exposure effect* first described by Zajonc [20], whereby repeated exposure to a specific stimulus improves an individual’s attitude toward it.

Substantial empirical evidence supports the existence of the mere exposure effect [32]. For instance, experiments have demonstrated that exposure to a stimulus increases the liking of it [20,32]. Also, repeated exposure to information about a firm affects employees’ identification with it and its perceived attractiveness [33]. The positive attitudinal change–increased liking–is robust across various stimuli and ratings [18].

Interestingly, familiarity with a stimulus does not require any conscious recognition of it [34]. Even unconscious exposure to a stimulus produces a positive effect through the process of positive reinforcement [18,20]. We applied the mere exposure logic to the evaluation of ideas and suggest that individuals’ familiarity with an idea improves their evaluation of it–a more familiar idea receives better evaluations. Ideas represent a stimulus that idea evaluators may or may not have encountered before. In the light of mere exposure research, decision-makers likely favor more familiar ideas, i.e. ideas they have already encountered, over ideas they have not yet been exposed to.

Several mechanisms can explain why familiarity is conducive to positive idea evaluations. First, processing fluency may be a cognitive pathway linking familiarity and favorable idea evaluation. As opposed to unknown ideas, more familiar ideas are more easily processed, meaning that individuals can process previously perceived stimuli faster. This increased fluency and reduced cognitive effort may then be misattributed as liking [35] or may directly result in liking [36]. Simply put, ideas that are easier to evaluate may be perceived as better.

The second explanation is that familiarity breeds certainty and, in turn, liking [37]. When a stimulus is first presented to an individual, it causes arousal owing to uncertainty about its potential consequences. With repeated exposure and higher familiarity, the individual learns about the likely outcomes, reducing uncertainty and enhancing liking. Again, this pathway may be relevant for idea evaluation such that familiarity with an idea reduces uncertainty; in turn, feelings of lower uncertainty engender more positive idea evaluation [23].

Finally, familiarity may lead to liking via social validation. If evaluators are exposed to a stimulus repeatedly, they may assume that it is more socially desirable [38]. Social desirability of an artifact or an idea can associated with increased liking of that artifact [27].

In sum, we expect that an individual’s degree of familiarity with an idea positively affects their evaluation of it. Thus:

**Hypothesis 1:** *Individuals are likely to assess ideas more positively if they are more familiar with them*.

## Method choice

In this paper, we use a hypothetico-deductive procedure to understand the relationship between familiarity and idea evaluation. Based on existing theory we developed an explanation of how and why familiarity affects idea evaluation, which we now proceed to test and validate using quantitative methods [39]. Method choice is driven by the state of theory in the field of inquiry [40]. Domains with nascent and immature theory lend themselves to qualitative research, as the discovery of new constructs and tentative relationships between them can be better achieved inductively. Domains with mature theory are better suited for quantitative methods since existing theory allows the deduction of testable hypotheses.

In this paper we rely on the mature and well established body of psychological literature on familiarity [16,17] and the so-called *mere exposure effect* [18–20] to establish a hypothesis–yielding quantitative methods as the means of choice for our investigation. Specifically, we use a factorial survey design (Studies 1 and 2) and a lab experiment (study 3) to validate our hypothesis.

## Study 1

### Study 1: Overview and methods

In study 1, we sought to mirror the idea evaluation process in organizations in a factorial survey design using ideas as vignettes to be evaluated. The sample included randomly selected participants from the engineering and marketing departments of a large car manufacturer (n = 210). The company management was aware of the data collection and allowed us to conduct the study but did neither interfere with the design of the study nor solicit any design changes. The company’s worker’s council also cleared the study to safeguard worker rights and interests. Participation was voluntary and anonymous; 69% of the participants were male, 7% had a management role, and most were between 30 and 40 years old.

Studies 1 and 2 did not go through an academic institutional review board for three reasons. First, we received ethical clearance from the workers’ council in which the study was conducted. Second, all data was anonymously collected and analyzed. We did not collect or store identifying information at any stage of the study. We did neither collect any sensitive information (e.g., religion, nationality, state of health) nor personal information (e.g., name, (e-mail) address, phone number) of the participants. Third, the department in which we were working at the time of the research process did not have an institutional review board and did not require us to go through ethics clearance for the type of research conducted.

Participants gave oral consent when taking part in the study. We instructed them that their responses would be analyzed in aggregate form and anonymously only. We also clarified that we collected the data for research purposes only. Their consent was documented by one of the researchers conducting the study. The surveys were collected after the study, but due to a random participation code, answers could not be linked with individuals.

In the experiment, participants evaluated 10 ideas and noted how familiar they felt with them. To minimize order effects, we randomized the sequence in which the ideas were presented. At 10 ideas per person, 10 x 210 = 2,100 measurements resulted.

In part 1 of the experiment, participants evaluated the 10 ideas in a randomized order and were asked to indicate how familiar they were with each (using a five-point Likert scale: *I am familiar with this idea*). We measured the dependent variable, idea evaluation, on a five-point Likert scale using the items from [41]: *It is very likely that we will take this idea to our supervisors*; *I think this idea should be implemented*; *I agree with this idea*; *This idea is valuable*. The reliability of this measure was high (Cronbach’s α = 0.93).

### Study 1: Material

We organized two workshops to come up with the ideas to be used in the study, one with six engineers from the focal organization to get an inside perspective, and the other with six PhD students so as to get an outside perspective. The workshops produced a set of 43 ideas, which we then clustered into topics. We merged similar ideas and then rated them in terms of their applicability to this study. We selected the 10 best-rated ideas, which covered five product categories and had a text length of approximately 50 words each. Prior to the main study, we performed a pre-test with 10 innovation managers to ensure that the ideas were realistic and that the questionnaire design was intuitive.

### Study 1: Results and discussion

Using this field data, we tested the prediction that higher familiarity with a given idea is associated with a better evaluation. As each evaluator assessed 10 ideas, we used fixed-effects regression to account for any unobserved variance in idea evaluators and ideas. Fixing the variance of ideas and evaluators, we found a positive and significant relationship between self-reported familiarity and idea evaluation (coefficient: β = 0.204, p = 0.000, overall model: F(217, 1,860) = 15.55, p = 0.000, R^2^ = 0.520, n = 2,078).

The results from the first correlational study support H1: Familiarity was positively associated with idea evaluation. However, since familiarity and idea evaluation were self-reported, not manipulated, we could not include a control group, which limits the generalization of the results. While we controlled for idea-specific effects in the analyses with idea-fixed effects, we could not rule out endogeneity rooted in confounders or omitted variables and thus cannot interpret our findings causally.

## Study 2

### Study 2: Overview and methods

Study 2 built on the same sample as study 1 and was conducted directly thereafter. In study 2, participants had to reevaluate one randomly selected idea out of our set of 10. In this part of the experiment, we measured evaluation with a single item: *This idea is valuable*. Our goal was to establish whether individuals evaluated ideas higher the second time they saw them, which would support our hypothesis.

### Study 2: Results and discussion

We used a paired t-test to check, in a within-subjects design, whether evaluators perceive the given idea as more valuable when they see it for the second time. We found that evaluators assigned a significantly higher value to the idea (M = 3.57, SD = 1.07) the second time compared to the first time (M = 2.59, SD = 1.40, t(206) = 10.35, p = 0.00). We take this as an indication of a familiarity effect.

In this study, we used the reevaluation of one of the ideas as a quasi-experimental manipulation of idea familiarity, which allowed us to replicate the familiarity effect in study 2. While the field experiments exhibit a high external validity, they may suffer from a lack of internal validity. For instance, we still had no control group, cannot preclude confounding factors’ influences, and cannot establish a causal effect. Thus, we replicated the results in a more controlled setting as follows.

## Study 3

### Study 3: Overview and methods

Building on our field study, we further tested whether we could establish a causal effect between familiarity and idea evaluation in a laboratory setting with high internal validity. Thus, we manipulated the familiarity of ideas in the lab.

Compared to the field studies, we adjusted the ideas for the lab experiment to be more relevant and appropriate to our student sample. Thus, while we used mobility ideas for the field experiment, here we used ideas regarding bicycles. Since the research question focused on familiarity–a basic psychological construct–we are confident that students represent an appropriate sample for replicating our study.

We performed the lab experiment at a German university. Participants were selected from a pool of students registered with the lab. The participant pool was invited via e-mail to the research lab and offered a reward of €12 per hour. The lab could accommodate 32 participants, and we organized multiple sessions per day; 285 students participated in the main experiment.

Study 3 did not go through an academic institutional review board for three reasons. First, we received ethical clearance from the academic lab in which the study was conducted. Second, all data was anonymously collected and analyzed. We did not collect or store identifying information at any stage of the study We did neither collect any sensitive information (e.g., religion, nationality, state of health) nor personal information (e.g., name, (e-mail) address, phone number) of the participants. Third, the department in which we were working at the time of the research process did not have an institutional review board and did not require us to go through ethics clearance for the type of research conducted.

The participant consent followed the standard procedures of the research lab in which the study was located. The participants consented to the study by agreeing to a consent form that had to be clicked before starting the experiment. We instructed them that their responses would be analyzed in aggregate form and anonymously only. We also clarified that we collected the data for research purposes only.

We used numbered place-cards to randomly assign every student to a computer. After entering a password that was provided at the start of the experiment, the instruction page informed the students that they had to rate a bike idea. Before starting the experiment, we introduced the participants to the study with the following text: *This experiment is about idea evaluation*. *You have a managerial role and are asked to evaluate the ideas you read*. *The ideas are randomly selected from a pool of ideas*. *Thus*, *you may see the same idea twice*. *Please wait until you are asked to leave the room*, *even if you finish ahead of time*. *The experiment takes around 25 minutes*.

We then used a procedure similar to the one in Harmon-Jones and Allen [36]: First, in part 1, the treatment was delivered. We exposed the participants to an idea without any action required. The exposure alone, even without conscious processing of the stimulus, is sufficient to create a familiarity effect. In the treatment condition, we induced familiarity by exposing the participants to the same idea in the exposure phase as in the measurement phase. In the no-treatment condition, we exposed the participants to a different but similarly valuable idea prior to the measurement phase.

In part 2, the participants were required to complete filler tasks meant to distract them without interfering with the manipulation [42]. We implemented a visual task (marking green spots on a picture), a verbal task (using anagrams), and mental calculations. Time was limited to two minutes per task to ensure that all the participants had the same amount of break time.

In part 3, we asked the participants to evaluate the idea (either the one that they had already been exposed to or a different one of similar value). We measured the dependent variable idea evaluation on a seven-point Likert scale using three items from [41]: *I think this idea should be implemented*; *I agree with this idea*; *This idea is valuable*. The participants then answered additional questions (e.g. demographics).

### Study 3: Material

The ideas that served as material for the experiment derived from a workshop with five researchers and consultants who are experts in technology management. In step 1, the group agreed to focus on bicycles as a context because students often use them. Next, we held an idea generation workshop, which resulted in 12 ideas. Third, these ideas were evaluated concerning novelty, feasibility, and overall value (a seven-point Likert scale) by 103 participants in the lab. In step 4, based on the students’ ratings, we adjusted the ideas until they were perceived to be similar in their overall value. Finally, we selected a subsample of four ideas for use in the main experiment. These ideas were very similar concerning their objective quality.

### Study 3: Results and discussion

Using regression with fixed effects on the idea level to account for inherent idea characteristics, we found a causal effect of familiarity in idea evaluation (coefficient: β = 0.436, p = 0.020, overall model: F(4, 278) = 5.21, p = 0.000, R^2^ = 0.070). The lab experiment’s results supported our hypothesis and presented causal evidence for a familiarity effect.

## Discussion

Idea evaluation is a crucial step in the innovation process. Understanding the factors that systematically influence evaluation outcomes beyond true quality is key to reducing evaluation errors. We found that familiarity (the opposite of subjective novelty) positively affects idea evaluation–individuals assess ideas more positively if they have been exposed to them before.

Our recent study is in line with existing research on the mere exposure effect, a psychological phenomenon where repeated exposure to a stimulus leads to a more positive attitude towards it [20,32]. Like previous researchers, we find that familiarity, achieved through repeated exposure to a stimulus, increases individuals’ positive evaluations. This reinforcement of earlier findings underscores the robustness of the mere exposure effect across different domains.

What our study adds to this body of knowledge is the application of the mere exposure effect to idea evaluation. We found that individuals evaluate ideas more favorably when they have been exposed to them before. This suggests that the mere exposure effect, previously studied in the context of objects, people, and organizations, extends to abstract concepts such as ideas.

Our recent study extends the body of knowledge on biases in the idea evaluation process, particularly focusing on how the familiarity of ideas influences their assessment. We found that individuals assess ideas more positively if they have been exposed to them before. We built on the body of knowledge on biases in the idea evaluation process, which has pointed out that the uncertain nature of the idea evaluation process renders idea evaluation inaccurate. Since the true value of an idea is unknown, evaluators rely on other cues that are available, but that may introduce error and bias into idea evaluation. Existing literature has investigated different factors that represent relevant (and biasing) cues in idea evaluation [15], e.g., characteristics of the idea creator [e.g., 24–26], the idea evaluator [e.g., 9,14,24,25,27,28], the idea evaluation context [e.g., 4,9,29,31], and the evaluation target [e.g. 2–4]. Our paper speaks to this last strand of research and demonstrates that familiarity, the opposite of subjective novelty, positively affects idea evaluation. This suggests that the mere exposure effect is applicable to idea evaluation processes, introducing a new perspective to the existing cues evaluators use.

### Implications for research

Our findings inform prior literature in several ways. First, we contribute to the research into idea evaluation in innovation in general [e.g. 2,9,15] by shedding light on familiarity (or its conceptual opposite, subjective novelty) as an independent driver of individuals’ evaluation decisions. Idea familiarity is likely to be ubiquitous in organizational innovation processes, since new ideas evolve over time and are likely to be discussed repeatedly in partly overlapping groups. This makes prior idea exposure a key variable that has to date been largely overlooked in the research.

Second, we add to the body of research that has focused on collective/objective novelty, which describes a relationship between an idea and a collective, such as a firm, a panel, a body of knowledge, or a social system [2,12,13]. Drawing on [10], we have extended the prevailing notion of novelty by highlighting the subjectivity of idea novelty or familiarity [9]. Subjective novelty describes the relationship between an idea and an individual idea evaluator; thus, it differs between individuals.

Third, our findings that more familiar ideas are less likely to be devalued than unfamiliar ideas also bears on literature whereby individuals tend to reject ideas if they feel uncertain in evaluation situations [23]. Following this literature, uncertainty reduction may be a principal mechanism whereby idea familiarity leads to increased liking.

Finally, we suggest that familiarity is an underappreciated mechanism that explains some well-known phenomena. For instance, the not-invented-here syndrome [5,43] may partly be driven by a familiarity effect, since individuals are more likely to be familiar with internal than with external ideas and therefore positively inclined toward the former and biased against the latter. Also, organizational myopia leading to the lack of ability to come up with breakthrough ideas may be partly rooted in the fact that decision-makers favor familiar ideas over unfamiliar ones [44].

### Implications for practice

Our study has important implications for practitioners. The bias toward familiar ideas that we have uncovered harms innovation success in organizations as it counteracts the goal to find, select, and implement highly novel ideas. Firms find it hard to overcome this bias, since it is often individual decision-makers who decide about the fate of ideas.

Based on our research, we advise that managers be more aware that they are subject to familiarity bias. It can be counteracted by putting evaluation panels in charge of particularly important decisions and by job rotation as it can offset the biasing effect of individual idea familiarity. Finally, distributed idea evaluation (e.g., internal crowdfunding [25]) is gaining in popularity as a new tool in the decision-making toolbox. It helps to overcome individual level familiarity biases, as long as subjective familiarity with an idea is differently distributed across evaluators: A more diverse group of evaluators is likely to have a broader range of familiarities with different ideas, reducing the overall bias in the decision-making process.

Viewed from a different angle, our findings also add to the toolbox of influence tactics [3], since employees can use sequences of prior exposure with ideas to convince supervisors of their ideas.

### Limitations and future research

This study has several limitations, which also open up directions for future research.

First, we did not consider boundary conditions to our findings. Familiarity and subjective novelty may have differential effects depending on other key variables. That is, we would expect that familiarity’s effects may depend on the context (e.g. high vs. low uncertainty), the idea type (e.g. ideas with high vs. low collective novelty), the idea source (e.g. is the ideator inside or outside the firm), and evaluator characteristics (e.g. high vs. low openness to new experiences). Future research could benefit from investigating these contingencies.

Second, we did not measure the de facto mediating process by which idea familiarity leads to higher idea evaluation. We shed light on a number of potential candidates that may drive idea familiarity’s effects on idea evaluation, such as fluency or reduced uncertainty. We encourage researchers to be more explicit about the respective path and to identify under what conditions each path is likely to operate.

Third, we have investigated familiarity’s effects for single exposures only. For repeated exposure, familiarity’s effects may weaken or may even reverse. When we entered the quadratic term of familiarity in our factorial survey study, we found significant decreasing returns for familiarity, but the effect remained positive over the full range of responses. Future research should investigate whether this positive effect turns negative and leads to reduced evaluation for very high exposure levels.

Fourth, the field studies were conducted within a large automotive firm, which might limit the generalizability of the results to other industries and organizations. Future research should replicate the study in different industries and organization types, which will help validate the findings and increase the generalizability of the results.

## Conclusion

In this paper, we argued that one reason for the failure of organizations to realize radical or disruptive ideas is due to the individuals responsible for innovation who may be tasked with finding novel ideas but who give preference to familiar ideas. We propose subjective novelty as a distinct concept and argue that individual idea evaluators are likely to be guided by their subjective perceptions of novelty or its counterpart, familiarity. Drawing on the rich psychological literature on familiarity and the mere exposure effect, we present two field studies and one laboratory study showing that familiarity with an idea positively affects the outcome of its evaluation, irrespective of true idea quality.
